# Morphometric Analysis of the Mandibular Canal in Van Cats: A Cone-Beam Computed Tomography (CBCT) Study

**DOI:** 10.3390/ani16193073

**Published:** 2026-09-29

**Authors:** Veysel Delibaş, Sema Kaya

**Affiliations:** 1 Department of Anatomy, Faculty of Veterinary Medicine, Van Yuzuncu Yil University, Van 65080, Türkiye; 2 Department of Oral and Maxillofacial Radiology, Faculty of Dentistry, Van Yuzuncu Yil University, Van 65080, Türkiye; semakaya@yyu.edu.tr

**Keywords:** anatomy, cone-beam computed tomography, mandibular canal, morphometric

## Abstract

This study aimed to determine the morphometric characteristics and sex-dependent variations in the mandibular canal (MC) in Van cats using Cone-Beam Computed Tomography (CBCT), thereby establishing breed-specific baseline values. CBCT scans of 14 healthy adult Van cats (7 males, 7 females; all aged 4 years old; mean body mass 3.3 ± 0.49 kg) were analyzed. DICOM files were oriented into a standardized head position by aligning the hard palate parallel to the horizontal plane and ensuring the sagittal plane was perpendicular to the reference line, and internal canal dimensions, cortical distances, ventral height (Bh), depth, and total mandibular dimensions were measured across eight standardized transverse planes using Multiplanar Reconstruction (MPR) software (Version 3.10, Carestream Dental LLC). Data were analyzed using Wilcoxon signed-rank and Mann–Whitney U tests. No statistically significant differences were found between right and left mandibles, demonstrating high bilateral symmetry. Similarly, internal canal dimensions also showed no significant differences by sex. However, in males, the canal ran significantly higher from the ventral border (Bh parameter), placing it closer to tooth roots. In both sexes, the MC ran closer to the lingual cortex in the molar region and shifted toward the vestibular cortex in the premolar region. Clinically, this high bilateral symmetry allows the intact side to serve as a reliable surgical reference in trauma cases. Buccal surgical approaches offer wider safety margins than lingual ones. Males Van cats carry a higher iatrogenic risk during dental procedures due to tooth root proximity, whereas female Van cats require a higher needle insertion point for inferior alveolar nerve blocks. In conclusion, this study provides a comprehensive, CBCT-based morphometric baseline of the mandibular canal in Van cats, offering essential data to enhance surgical precision and minimize iatrogenic risks during feline maxillofacial procedures.

## Simple Summary

Although Cone-Beam Computed Tomography (CBCT) is established in veterinary dentistry for evaluating feline maxillofacial structures and dental abnormalities, comprehensive multi-landmark morphometric analyses of the canalis mandibularis in cats remain limited. Using the Van cat as a model, this study addresses this gap by providing a detailed evaluation of the canal’s trajectory along the entire length of the mandible. Unlike previous canine and feline studies that restricted their scope to isolated regions or single root apices, this research introduces a broader approach by analyzing eight distinct anatomical landmarks, extending from the foramen mandibulare to the rostral foramen mentale. This approach yields a precise morphometric mapping of the mandibular canal trajectory. Ultimately, these findings aim to provide an enhanced anatomical blueprint and clinical reference for veterinary surgeons, thereby reducing the risk of neurovascular injury during critical feline maxillofacial procedures, including tooth extractions, endodontic treatments, and fracture fixations.

## 1. Introduction

The mandibular canal (MC) is a critical intraosseous passage within the mandible that transmits the inferior alveolar neurovascular bundle, providing sensory innervation and vascular supply to the teeth, gingivae, and adjacent soft tissues [1,2]. In carnivores, the canal originates at the foramen mandibulare, located on the medial surface of the ramus mandibulae adjacent to the pterygoid fossa the insertion site of the musculus pterygoideus medialis [3]. It courses rostrally within the mandibular body and opens onto the buccal surface via the mental foramina (rostral, middle, and caudal/distal) at the level of the canine and premolar teeth [3].

In dentoalveolar surgery, precise knowledge of the spatial relationship between the MC and adjacent tooth roots is crucial [4]. Oral and maxillofacial pathologies are highly prevalent in feline practice, with tooth extraction representing the most frequently performed surgical procedure [5]. During extractions and other endodontic or oncologic surgical interventions, improper technique or anatomical blind spots can lead to severe iatrogenic complications, including profuse hemorrhage, mandibular fractures, root/tooth displacement, and permanent nerve injury [6,7,8]. Consequently, preserving the structural integrity of the MC and its neurovascular contents is paramount during surgical procedures involving the feline lower jaw [9,10,11].

Cone-Beam Computed Tomography (CBCT) enables precise visualization of complex maxillofacial structures and abnormalities through multiplanar reconstruction (MPR) and three-dimensional (3D) modeling capabilities [12,13,14]. This modality represents a validated, reliable tool for sub-millimetric spatial assessment of the MC prior to surgical interventions [15,16]. The Van cat is a breed originating in Turkey, characterized by a triangular-shaped head, small nose, and body entirely covered with white hair, with its most distinctive feature being its characteristic eye coloration, with one eye typically blue and the other light amber-yellow [17].

While the extensive literature in dentistry and maxillofacial surgery has characterized mandibular osteology in humans [18,19] and dogs [10,12,20], comparable morphometric research regarding domestic or wild felids remains remarkably limited [21,22]. Although the application of CBCT in feline diagnostic imaging has been documented in a few studies [23,24], standardized quantitative baselines for regional mandibular morphometrics are still lacking. In felids, various painful dental conditions requiring surgical management such as tooth resorption can lead to severe craniofacial dysfunction [6]. Consequently, a lack of detailed anatomical knowledge during surgical planning increases the risk of severe iatrogenic complications [11,25]. Therefore, the primary objective of this study was to quantitatively evaluate the morphometric variations in the mandibular canal in male and female Van cats using CBCT, thereby establishing breed-specific anatomical baselines to enhance clinical safety during feline maxillofacial procedures.

## 2. Materials and Methods

This study was approved by the Local Ethics Committee for Animal Experiments (decision number: 2026/07-25). A total of 14 healthy adult Van cats (7 males, 7 females) were evaluated based on high-quality Cone-Beam Computed Tomography (CBCT) scans. Systemic health status was confirmed through a comprehensive physical examination (assessment of body temperature, hydration status, mucous membrane color, and cardiopulmonary auscultation) alongside a complete medical history showing no evidence of chronic disease or recent pharmacological intervention. Inclusion criteria comprised intact (non-neutered) adult cats, exactly 4 years of age, with a mean body mass of 3.3 ± 0.49 kg, displaying no visible anatomical anomalies or prior surgical interventions. Conversely, the exclusion criteria comprised juvenile individuals with incomplete dental development, geriatric cats, and any subjects presenting with dental or periodontal diseases.

**Figure 1:**
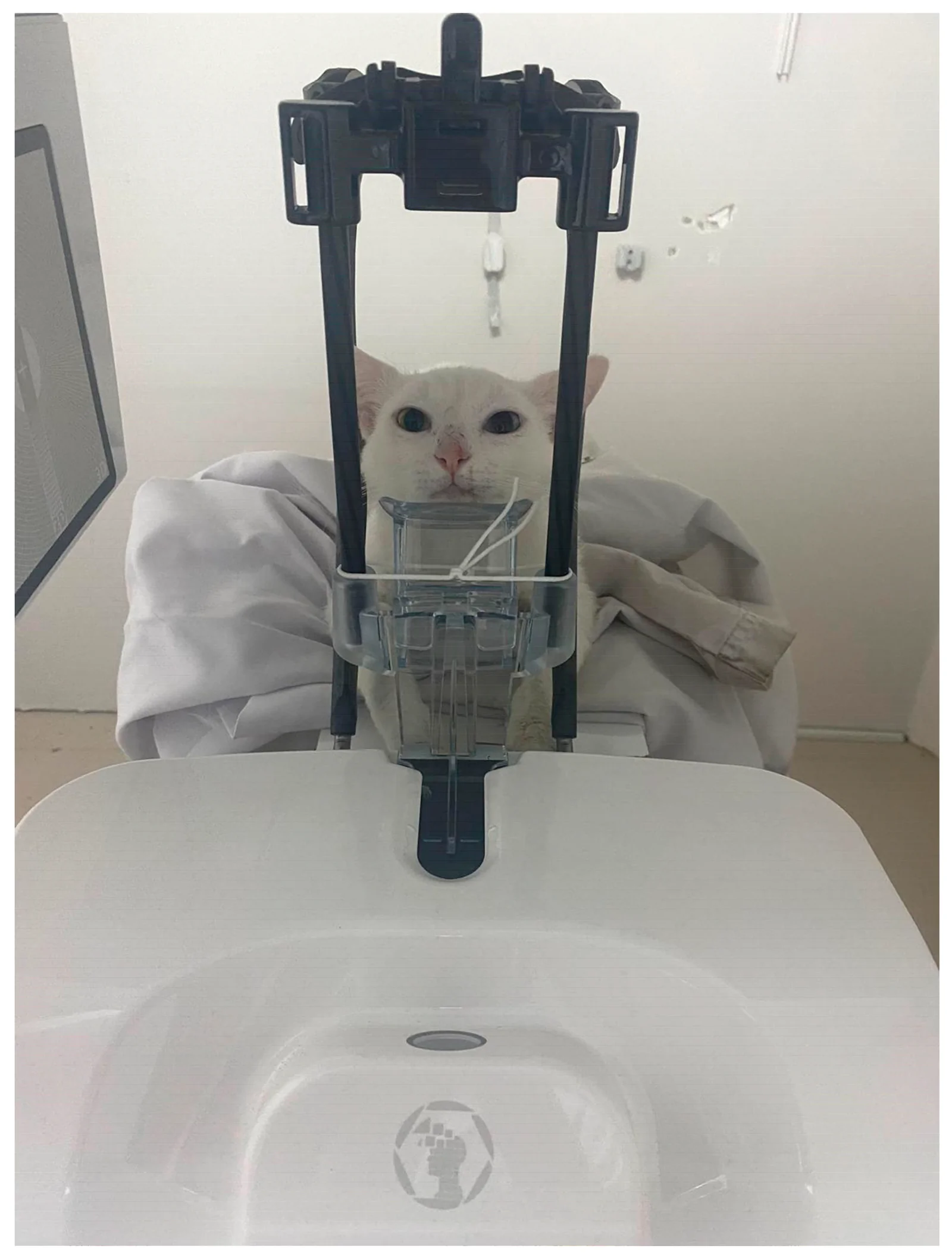
A Van cat secured for imaging in a CBCT machine.

To prevent motion artifacts and ensure complete immobility during CBCT acquisition, a standardized sedation protocol was applied. Food and water were withheld for 8 h and 3 h, respectively, prior to sedation to minimize the risk of aspiration pneumonia. Sedation was induced via intramuscular (IM) administration of xylazine hydrochloride(at a dose of 1–2 mg/kg body weight; Alfazyne^®^ 2% injectable solution, Ege Vet Veterinary Medicines, İzmir, Türkiye). Depth of sedation and vital signs were monitored throughout the procedure. Prior to imaging, a thorough oral examination was performed on all cats. The CBCT imaging was conducted using the scanner located at the Faculty of Dentistry. Each cat was positioned in sternal recumbency on a height-adjustable scanning table, with the head extended onto a carbon-fiber, radiolucent extension platform specifically designed for the scanner (Figure 1). To ensure reproducible head alignment and minimize motion artifacts, positioning foam wedges were placed beneath the neck, centering the head within the CBCT field of view (FOV) (Figure 1).

**Figure 2:**
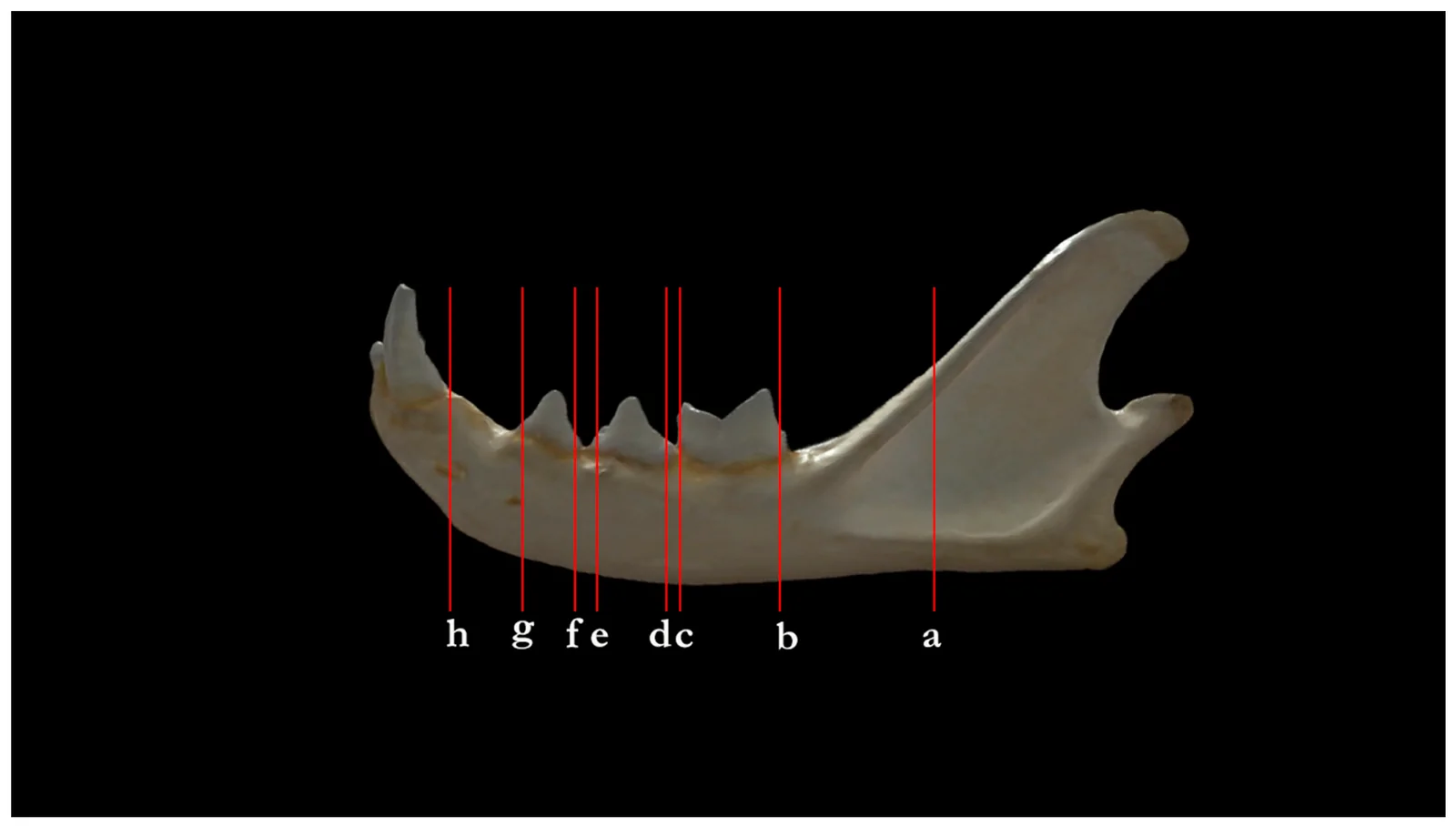
Schematic representation of the eight transverse plane landmarks used for morphometric evaluation of the mandibular canal. (a) F mand (foramen mandibulare): transverse plane passing through the foramen mandibulare; (b) RD M1 (distal root of first molar): transverse plane passing through the distal root aspect of the first molar tooth; (c) RM M1 (mesial root of first molar): transverse plane passing through the mesial root aspect of the first molar tooth; (d) RD PM2 (distal root of second premolar): transverse plane passing through the distal root aspect of the second premolar tooth; (e) RM PM2 (mesial root of second premolar): transverse plane passing through the mesial root aspect of the second premolar tooth; (f) RD PM1 (distal root of first premolar): transverse plane passing through the distal root aspect of the first premolar tooth; (g) RM PM1 (mesial root of first premolar): transverse plane passing through the mesial root aspect of the first premolar tooth; (h) F ment (rostral foramen mentale): transverse plane passing through the rostral foramen mentale.

**Figure 3:**
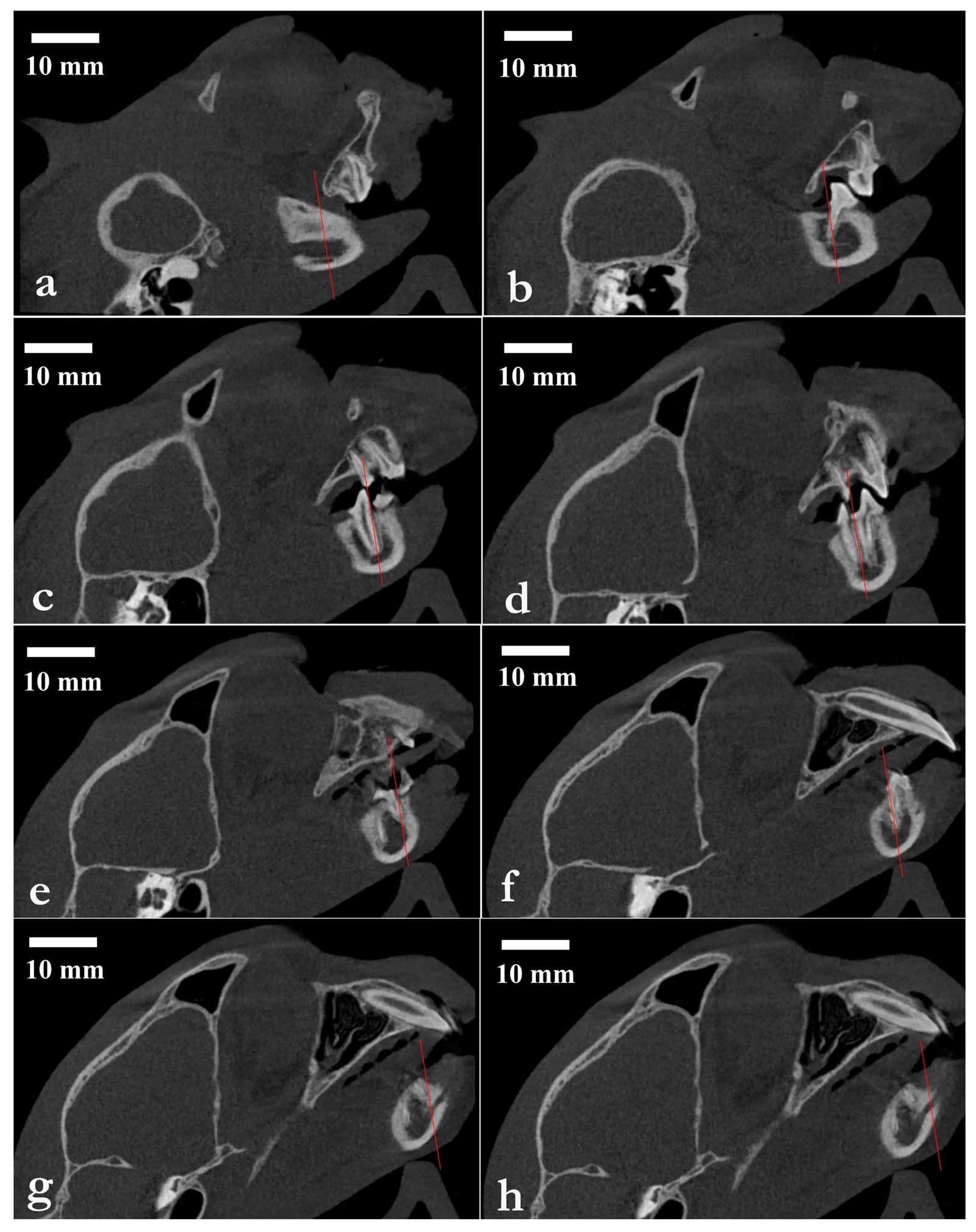
CBCT sagittal image of eight measurement sites (red line) used in morphometric measurements of the mandibular canal. (**a**) F mand (foramen mandibulare): transverse plane passing through the foramen mandibulare; (**b**) RD M1 (distal root of first molar): transverse plane passing through the distal root aspect of the first molar tooth; (**c**) RM M1 (mesial root of first molar): transverse plane passing through the mesial root aspect of the first molar tooth; (**d**) RD PM2 (distal root of second premolar): transverse plane passing through the distal root aspect of the second premolar tooth; (**e**) RM PM2 (mesial root of second premolar): transverse plane passing through the mesial root aspect of the second premolar tooth; (**f**) RD PM1 (distal root of first premolar): transverse plane passing through the distal root aspect of the first premolar tooth; (**g**) RM PM1 (mesial root of first premolar): transverse plane passing through the mesial root aspect of the first premolar tooth; (**h**) F ment (rostral foramen mentale): transverse plane passing through the rostral foramen mentale.

**Figure 4:**
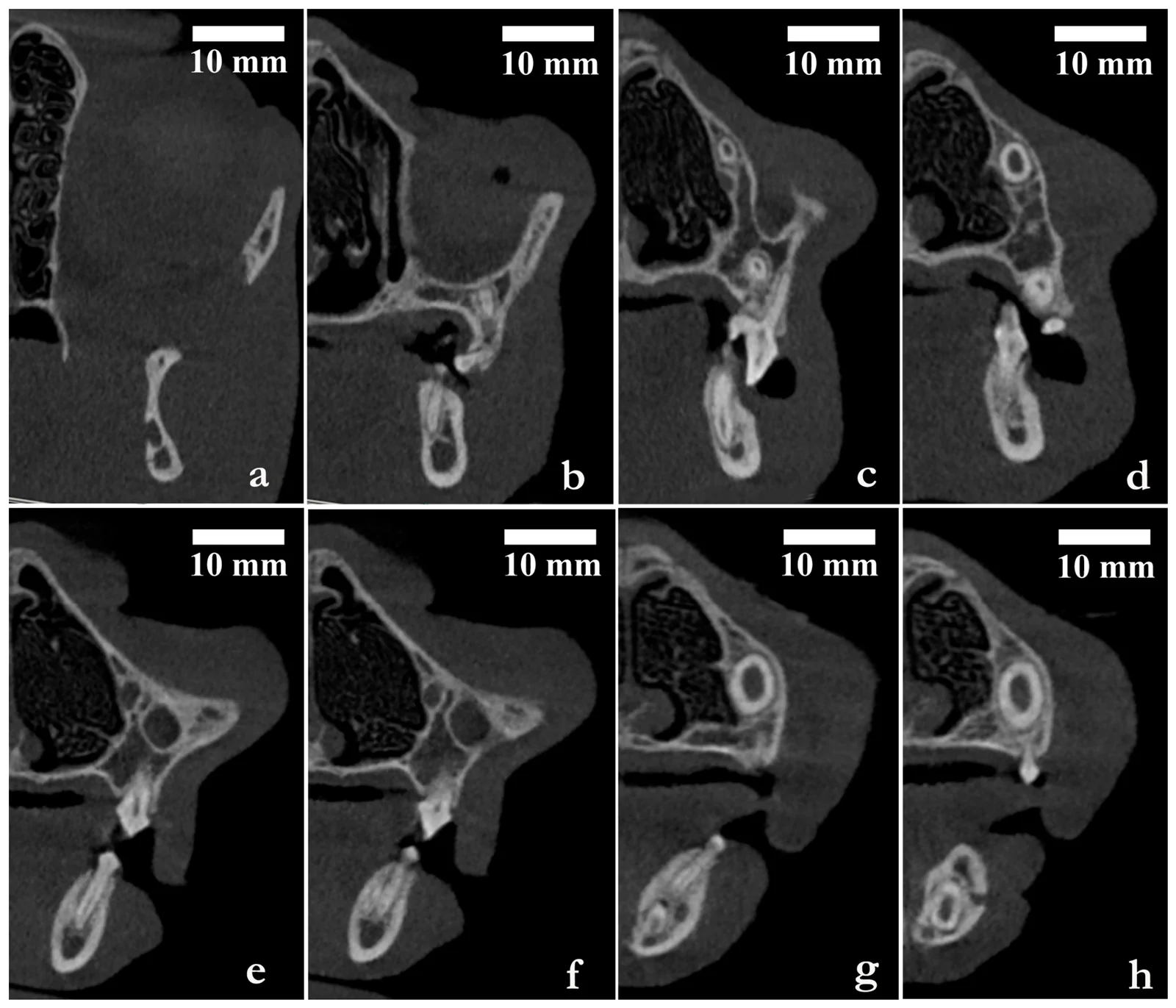
CBCT transversal image of eight measurement sites used in morphometric measurements of the mandibular canal. (**a**) F mand (foramen mandibulare): transverse plane passing through the foramen mandibulare; (**b**) RD M1 (distal root of first molar): transverse plane passing through the distal root aspect of the first molar tooth; (**c**) RM M1 (mesial root of first molar): transverse plane passing through the mesial root aspect of the first molar tooth; (**d**) RD PM2 (distal root of second premolar): transverse plane passing through the distal root aspect of the second premolar tooth; (**e**) RM PM2 (mesial root of second premolar): transverse plane passing through the mesial root aspect of the second premolar tooth; (**f**) RD PM1 (distal root of first premolar): transverse plane passing through the distal root aspect of the first premolar tooth; (**g**) RM PM1 (mesial root of first premolar): transverse plane passing through the mesial root aspect of the first premolar tooth; (**h**) F ment (rostral foramen mentale): transverse plane passing through the rostral foramen mentale.

All Cone-Beam Computed Tomography (CBCT) scans were acquired using a CS 9600 system (Carestream Dental LLC, Atlanta, GA, USA) with an 8 × 5 cm mandibular field of view (FOV). Exposure and acquisition parameters were standardized across all subjects as follows: tube voltage of: 120 kVp, tube current-time product of 5 mAs, scan duration of 7 s, and an isotropic voxel size of 0.4 mm. Image analysis and morphometric evaluations were performed using CS 3D Imaging software (Version 3.10, Carestream Dental LLC). Prior to morphometric measurements, DICOM datasets were transferred to a dedicated workstation and reoriented into a standardized head position using the Multiplanar Reconstruction (MPR) module (Version 3.10, Carestream Dental LLC). The Frankfort horizontal plane, defined by the bilateral orbitale and porion points, was adjusted parallel to the floor, and the mid-sagittal plane was aligned with the facial midline. Morphometric evaluation of the canalis mandibularis along its rostrocaudal trajectory was conducted across eight standardized anatomical landmarks. The definitions and spatial locations of these reference landmarks are illustrated in Figure 2, Figure 3 and Figure 4.

**Figure 5:**
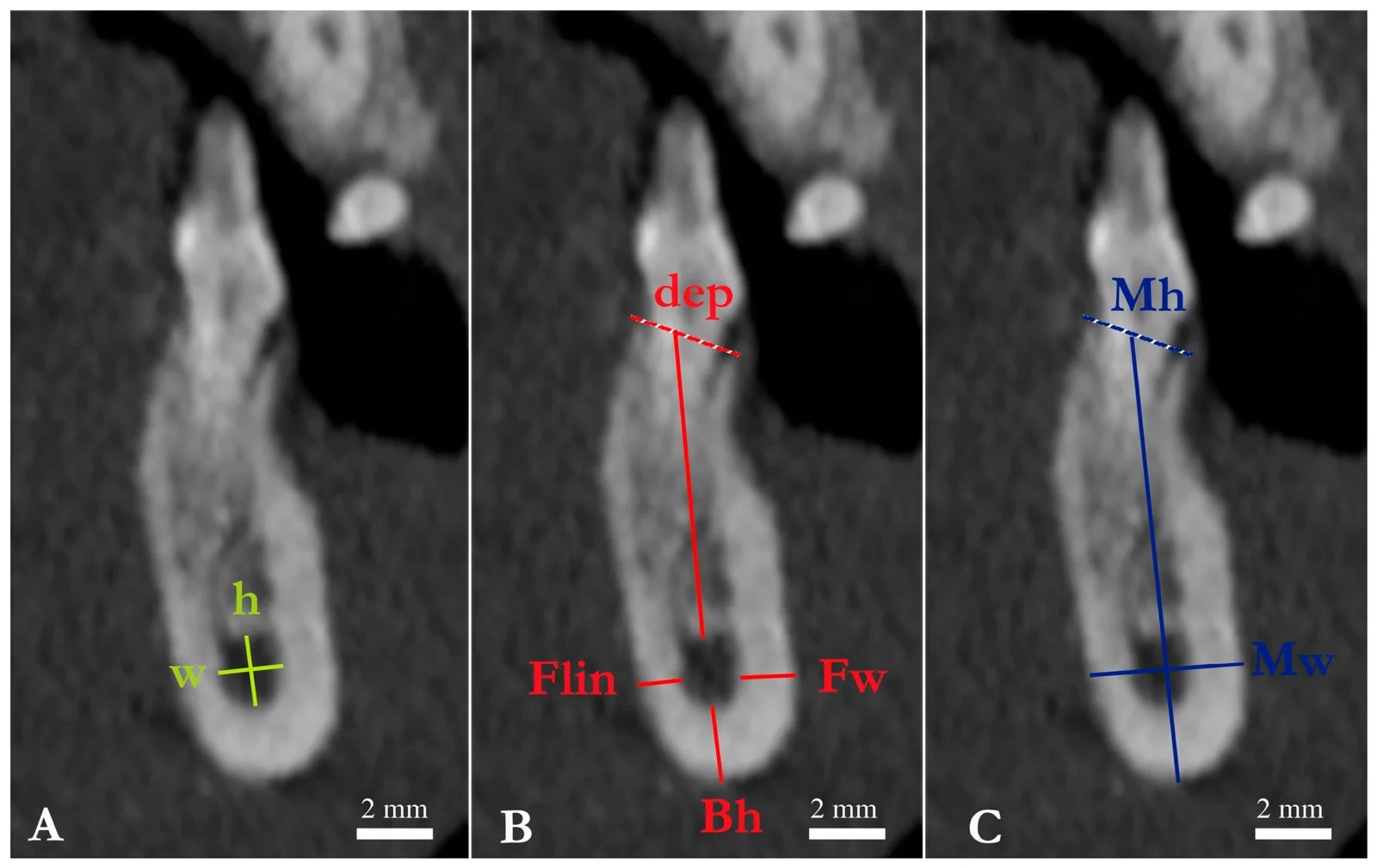
Morphometric measurement parameters of the mandibular canal. (**A**) h: height of the canalis mandibularis, w: width of the canalis mandibularis; (**B**) Flin: distance between the canalis mandibularis and the lingual border, Fw: distance between the canalis mandibularis and the vestibular border, Bh: distance from the canalis mandibularis to the ventral border, dep (depth of the mandibular canal): the vertical distance measured from the alveolar crest line (proximal reference point, dashed red line) to the superior border of the mandibular canal; (**C**) Mh (total vertical height of the mandible): the distance measured from the most superior point of the alveolar crest (proximal reference point, dashed blue line) to the base of the mandible, Mw: width of the mandible.

To ensure measurement consistency and anatomical accuracy, all CBCT evaluations were performed by an experienced veterinarian trained in diagnostic imaging. Quantitative parameters including the internal dimensions of the mandibular canal (Figure 5A), its spatial relationships relative to the external cortical borders (Figure 5B), and the overall mandibular height and width (Figure 5C) were recorded at each predefined anatomical reference landmark:

h: Height of the canalis mandibularis (Figure 5A).

w: Width of the canalis mandibularis (Figure 5A).

Flin: Distance between the canalis mandibularis and the lingual border (Figure 5B).

Fw: Distance between the canalis mandibularis and the vestibular border (Figure 5B).

Bh: Distance from the canalis mandibularis to the ventral border (Figure 5B).

dep (Depth of the mandibular canal): The vertical distance measured from the alveolar crest line to the superior border of the mandibular canal. (Figure 5B).

Mh (Total vertical height of the mandible): The distance measured from the most superior point of the alveolar crest to the base of the mandible. (Figure 5C).

Mw: Width of the mandible (Figure 5C).

The normality of continuous variables was assessed using the Shapiro–Wilk test. Because the datasets did not follow a normal distribution, non-parametric statistical methods were employed. Paired morphometric parameters between the right and left mandibles were analyzed using the Wilcoxon signed-rank test to evaluate bilateral symmetry. Differences between sexes (male vs. female) were evaluated using the Mann–Whitney U test. Continuous variables are expressed as “mean ± standard deviation (SD)” All statistical analyses were performed using SPSS software (version 21.0; IBM Corp., Armonk, NY, USA). A two-tailed *p* value < 0.05 was considered statistically significant.

## 3. Results

Morphometric analysis revealed distinct spatial variations in mandibular canal (MC) height along its rostrocaudal trajectory. In female Van cats, the maximum MC height was observed at the level of the foramen mandibulare. The height exhibited a marked decrease toward the mesial root of the first molar tooth and remained relatively constant up to the distal aspect of the second premolar. It then elevated rostrally toward the mesial root of the first premolar before progressively tapering toward its junction with the rostral foramen mentale, which represented the point of minimal canal height (Table 1 and Table 2). Although a similar overall trend was observed in male Van cats, the MC maintained a noticeably more uniform height extending from the mesial aspect of the first molar tooth to the mesial aspect of the second premolar (Table 1 and Table 2).

Similarly to the height parameters, the MC width exhibited marked spatial variations along its rostrocaudal trajectory (Table 1 and Table 2). In female Van cats, the canal maintained a broad diameter at its origin at the foramen mandibulare, attained its maximum width at the mesial root region of the first molar, and narrowed significantly at the mesial root region of the second premolar. From this landmark rostrally, the MC demonstrated continuous expansion up to the mesial root region of the first premolar, followed by a progressive narrowing toward its junction with the rostral foramen mentale, where the minimum width was recorded. In male Van cats, conversely, the canal was wide at the foramen mandibulare, reached its peak diameter at the distal root region of the first molar, and constricted at the mesial root region of the first molar. Thereafter, it displayed a steady expansion toward the mesial root region of the first premolar before tapering to its minimum width at the rostral foramen mentale junction.

The Fw and Flin values recorded along the course of the MC are summarized in Table 1 and Table 2. As the canal becomes embedded within the osseous parenchyma in the molar and premolar regions, the Flin value increases rapidly, indicating a central trajectory within the mandibular body. In both sexes, the MC progresses from the molar region toward the distal aspect of the first premolar near the exact center of the bone, maintaining a balanced trajectory with a slight lingual inclination. However, compared with females, the canal in males exhibits a trajectory positioned noticeably closer to the vestibular cortex, particularly at the level of the distal aspect of the second premolar. Moving rostrally, immediately prior to its exit through the rostral foramen mentale, the canal demonstrates a critical constriction at the level of the mesial root of the first premolar tooth. In this region, the MC shifts distinctly inward and toward the vestibular side in both male and female Van cats. Finally, at the level of the rostral foramen mentale, the Fw value reaches zero (Fw = 0.00) as the canal opens exteriorly through the vestibular cortex.

In our study, the Fw and Flin values for all Van cats were compared statistically. No overall difference was detected between Fw and Flin measurements across all foramen and tooth regions (*p* > 0.05), primarily due to the high variance introduced by zero (0.00) anatomical values. However, when these absolute zero values were excluded to specifically analyze the intraosseous course of the mandibular canal along the dental roots, Fw values were significantly greater than Flin values (Wilcoxon signed-rank test, *p* = 0.0067).

Furthermore, statistical analysis comparing the right and left canalis mandibularis parameters across all evaluated Van cats revealed no significant bilateral differences (Table 3), confirming a high degree of bilateral symmetry.

In terms of depth relative to the alveolar margin, the mandibular canal (MC) in Van cats was found to follow a slightly curved, U-shaped course rather than a linear trajectory. Furthermore, the depth of the MC was not uniform along its course, fluctuating in both female and male Van cats (Table 1 and Table 2). Similarly, the height and width of the mandible along the course of the MC were not uniform, exhibiting local increases and decreases (Table 1 and Table 2). The distal root region of the first molar tooth was recorded as the highest point of the mandibular body. Narrowing dorsoventrally at the mesial root of the same tooth, the mandibular body maintained a nearly constant height up to the mesial root of the first premolar tooth, exhibiting a slight elevation in the rostral mental foramen region. The widest point of the mandibular body was located in the mesial root region of the second premolar tooth, whereas the narrowest point was observed in the region corresponding to the mesial root of the first premolar tooth.

Regarding the statistical comparison of the mandibular canal between sexes, canal dimensions, specifically h, w, Fw, Flin, Mh, and Mw, showed no significant differences between female and male Van cats. However, the mandibular canal exhibited distinct sex-related variations along its topographic course (Table 4). Specifically, the distance from the mandibular canal to the ventral border (Bh) was significantly greater in male Van cats, positioning the canal higher relative to female Van cats. Conversely, the depth of the mandibular canal (dep) at the distal root of the first premolar tooth was significantly greater in females.

## 4. Discussion

In this study, the morphometric values of the mandibular canal (MC) in Van cats were determined for the first time using Cone-Beam Computed Tomography (CBCT). Diagnostic imaging modalities such as radiography, computed tomography, or magnetic resonance imaging are routinely utilized in the anatomical evaluation of the MC [26]. Computed Tomography (CT) is considered the gold standard for imaging hard tissues in dentistry due to its measurement accuracy and clear visualization of anatomical boundaries and variations [27]. Furthermore, it serves as a non-invasive, rapid, and highly reliable diagnostic tool with substantial clinical value [26]. In jawbone evaluation, tomographic imaging offers distinct advantages over two-dimensional methods by allowing multiplanar visualization (sagittal, transverse, and dorsal) of topographic relationships and the reconstruction of three-dimensional structures, thereby enhancing surgical planning [28,29]. One of the most critical parameters in the application of CT and CBCT in veterinary dentistry is spatial resolution, which is directly influenced by slice thickness [30,31]. In the present study, acquiring CBCT images at a 0.4 mm voxel size with an 8 × 5 cm mandibular FOV using the CS 9600 system (Carestream Dental LLC, Atlanta, GA, USA) provided adequate spatial resolution for precise morphometric evaluation. This and similar high-resolution protocols enable clear cross-sectional imaging of the MC without structural superimposition from adjacent anatomical structures [27].

In line with the MC diameter (height and width) results obtained in our study, it was determined that the canal height in Van cats followed a cycle of gradual decrease, remaining constant, increasing, and decreasing again along the dental arch, starting from the foramen mandibulare. Furthermore, the MC width in Van cats exhibited slight expansions and narrowings along its course. In this respect, our findings are consistent with those reported by Cotrim et al. (2015) [22] in mixed-breed cats, who similarly observed regional fluctuations in MC height along the dental arch as well as subtle variations in canal width at specific tooth locations. In a study conducted on humans, Scomparim (2013) [32] suggested that these slight fluctuations exhibited by the MC width along its course arise from the anatomical pressure created by the differing root sizes of the premolar and molar teeth. The maximum height (h) and width (w) of the mandibular canal (MC), measured at the level of the mandibular foramen, have significant practical implications for local anesthesia procedures. Knowledge of these dimensions at the mandibular entry point can facilitate effective anesthetic diffusion during tooth extractions and rostral or caudal mandibular interventions, while minimizing the risk of direct needle trauma or intraneural injection. The pronounced structural narrowing and inward shift in the MC observed at the mesial root of the first premolar, followed by its minimum height and width at the rostral foramen mentale junction, represent a high-risk zone during dental procedures. When performing complex extractions of the first premolar or surgical root tip retrieval, clinicians must exercise extreme caution.

When the proximity of the MC pathway to the lingual and vestibular surfaces was examined, it was revealed that the canal in Van cats followed a line close to the lingual surface up to the mesial aspect of the first molar tooth. From this point onward, it progressed centrally though still inclined toward the lingual direction up to the distal aspect of the first premolar tooth, and finally directed toward the vestibular margin from the first premolar tooth toward the rostral foramen mentale to open outward. Accordingly, our results demonstrate similarity to the findings of Cotrim et al. (2015) [22], who likewise reported that the mandibular canal in mixed-breed cats travels closer to the lingual cortical plate in the molar region before shifting toward the vestibular surface as it approaches the rostral foramen mentale. The general trajectory of this course also resembles the canal route in mesocephalic and brachycephalic dogs, where it runs close to the lingual surface in the region of the molar teeth, is subsequently centered between the vestibular and lingual cortices up to the third premolar, and terminates close to the vestibular cortex in the foramen mentale region [10]. Since the canal in Van cats is located closer to the lingual surface throughout most of the length of the mandibular body, it can be argued that it also shares similarities with the topography described in humans [18,19]. From a clinical standpoint, the regional course of the canal close to the medial (lingual) wall at the level of the molar teeth, and its direction from the midline toward the lateral (facial/vestibular) side at the premolar level in Van cats, provide crucial practical guidelines for veterinary dental and maxillofacial interventions. During mandibular fracture fixation (e.g., plate-screw applications), endodontic procedures, complex tooth extractions, or implant surgeries, manipulation of the mandibular body carries an inherent risk of damaging the inferior alveolar neurovascular bundle [19]. Our morphometric and statistical comparisons demonstrated that the lateral (facial) bone wall provides a significantly wider safe surgical margin compared to the lingual aspect. Consequently, surgical approaches targeting the lateral surface present a clinically safer trajectory to minimize potential neurovascular trauma during hardware placement or bone manipulation.

The absence of significant bilateral differences in our findings suggests that the topographic orientation of the mandibular canal (MC) in Van cats exhibits relatively stable and symmetrical morphometrics. While functional asymmetries, such as unilateral mastication or localized biomechanical forces, could theoretically influence bone remodeling, our cross-sectional morphometric data did not indicate any lateral bias. However, to definitively conclude that the morphology of the canal is entirely immune to long-term functional adaptation or biomechanical stress, further prospective studies investigating specific clinical variables, including age, sex, body mass, and functional chewing patterns, are warranted. The finding that the parameters of the canal particularly the distance to the ventral border (Bh) and mandibular width (Mw) did not differ significantly between the right and left sides presents a major practical advantage for veterinary surgeons. In cases of mandibular fractures, radiological or morphometric data obtained from the intact side can serve as a reliable reference for surgical planning, such as screw length selection and plate placement, on the damaged contralateral side. Nevertheless, this should not diminish the necessity for further studies aimed at characterizing the MC in other cat breeds with different cephalic biotypes, as suggested by Valarelli et al. (2007) [33]. Indeed, in this breed specific study on Van cats, it should be noted that during premolar and molar surgeries or endodontic procedures in male cats, the canal is located significantly closer to the tooth roots; this proximity may increase the risk of iatrogenic injury or root lesions exerting pressure on the canal. Conversely, when performing inferior alveolar nerve blocks at the level of the mandibular foramen in female cats, the needle insertion point may need to be adjusted relative to the ventral margin of the mandible. These anatomical variations appear to be closely associated with breed characteristics and overall morphological structure. As noted by Martinez et al. (2009) [10], such conditions can primarily be attributed to variations in the general body size of the animals analyzed.

When the depth (dep) of the mandibular canal (MC) was analyzed, our results partially differed from the values reported for mixed-breed cats [22]. In mixed-breed cats, as well as in brachycephalic and mesocephalic dogs, the canal reportedly descends from the mandibular foramen to the mesial root region of the first molar tooth, where it reaches its maximum depth, and displays a linear, smooth ascent toward the rostral mental foramen [22]. In contrast, our findings in Van cats indicate that this path does not follow a simple linear trajectory, as the canal deepens again at the mesial aspect of the first premolar tooth. Martinez et al. (2009) [10] attributed the deepest position of the MC at the mesial aspect of the first molar tooth to the prominent height of its root within the mandibular body. Consequently, it can be hypothesized that the mesial root height of the first premolar tooth in Van cats may be relatively greater than that of the adjacent premolars. Nevertheless, to confirm this potential relationship, future investigations incorporating quantitative correlation analyses between precise MC coordinates and tooth root morphometrics are warranted. Furthermore, the observed vertical fluctuations along the MC trajectory may also be influenced by regional variations in the cortical bone architecture of the mandibular body. Supporting this structural role, Eto et al. (2023) [34] reported that buccal cortical bone thickness and height vary significantly by sex, with females exhibiting thinner cortical bone and reduced bone height relative to the position of the MC. Therefore, evaluating the relationship between these cortical bone variations and the MC alongside fundamental biological variables such as age, sex, body weight, and bilateral symmetry remains an important objective for future studies. The non-linear, U-shaped vertical trajectory of the MC along the mandibular body indicates that the risk of neurovascular involvement varies dynamically along the dental arch. Because the canal deepens at the molar region, rises rostrally, and deepens again at the first premolar tooth, standard linear assumptions of canal depth can lead to intraoperative complications. Surgical stabilization of mandibular body fractures must account for these vertical fluctuations to avoid impinging upon the canal during reduction and hardware engagement. Furthermore, the statistically significant sex-specific variations observed in our study dictate tailored surgical planning; female cats exhibited a significantly greater canal depth (dep) at the distal root of the first premolar tooth. These morphological distinctions suggest that clinicians should adjust drill depth and screw length based on sex when operating on the ventral mandibular border or placing hardware in the rostral premolar region in Van cats.

The primary limitations of this study include the relatively small sample size and the restricted demographic diversity of the study group, as well as the lack of cortical bone thickness measurements. The limited number of animals restricts the direct generalization of the obtained morphometric data to broader feline populations. Furthermore, because all animals evaluated were standardized at 4 years of age and exhibited a narrow body weight range, age-related changes (e.g., in young or geriatric individuals) and body weight-dependent variations in mandibular bone density, cortical thickness, or canal topography could not be evaluated. How marked variations in skull size or body weight influence the millimetric position of the MC remains to be determined in future studies. Finally, although vertical and horizontal canal fluctuations may be linked to regional variations in the ventral and lateral cortical bone, cortical bone thickness was not metrically quantified in the current study.

## 5. Conclusions

In conclusion, this study has revealed the topography of the mandibular canal and sex-dependent morphometric variations in Van cats with millimeter precision. The morphometric reference values obtained from this study will serve as a valuable guide for clinicians to prevent iatrogenic neurovascular damage during dental procedures and regenerative jaw surgeries in Van cats. Furthermore, we believe that the results of this study will pave the way for future large-scale population studies focusing on the mandibular canal, encompassing different cat breeds, age groups, weights, and cortical bone thickness measurements.

## Figures and Tables

**Table 1:** Mandibular canal measurement values of female Van cats (*n* = 7).

Measurements	h	w	Fw	Flin	Bh	dep	Mh	Mw
References	Mean ± SD (mm)	Mean ± SD (mm)	Mean ± SD (mm)	Mean ± SD (mm)	Mean ± SD (mm)	Mean ± SD (mm)	Mean ± SD (mm)	Mean ± SD (mm)
Right Mandibular Canal								
F mand	2.38 ± 0.33	2.18 ± 0.15	2.20 ± 0.13	0.00 ± 0.00	4.68 ± 0.58	-	-	4.39 ± 0.15
RD M1	1.93 ± 0.22	1.63 ± 0.25	1.40 ± 0.16	1.50 ± 0.22	2.48 ± 1.01	5.78 ± 0.25	10.19 ± 0.63	4.56 ± 0.29
RM M1	1.75 ± 0.31	1.81 ± 0.21	1.60 ± 0.27	1.55 ± 0.44	2.03 ± 0.33	5.93 ± 0.90	9.72 ± 0.88	4.97 ± 0.36
RD PM2	1.78 ± 0.35	1.73 ± 0.22	1.73 ± 0.43	1.38 ± 0.24	2.05 ± 0.31	5.70 ± 0.82	9.53 ± 0.57	4.85 ± 0.47
RM PM2	2.05 ± 0.41	1.65 ± 0.26	1.73 ± 0.33	1.68 ± 0.62	2.33 ± 0.87	5.53 ± 0.98	9.91 ± 0.66	5.07 ± 0.26
RD PM1	2.15 ± 0.25	1.68 ± 0.29	1.65 ± 0.39	1.33 ± 0.32	1.78 ± 0.37	5.85 ± 0.25	9.79 ± 0.49	4.68 ± 0.48
RM PM1	2.35 ± 0.41	1.80 ± 0.34	1.40 ± 0.14	1.03 ± 0.17	1.50 ± 0.41	6.05 ± 0.87	9.90 ± 0.68	4.25 ± 0.17
F ment	0.78 ± 0.19	0.81 ± 0.24	0.00 ± 0.00	3.70 ± 0.62	4.65 ± 0.70	4.75 ± 0.51	10.18 ± 0.30	4.53 ± 0.51
Left Mandibular Canal								
F mand	2.45 ± 0.33	2.25 ± 0.17	2.23 ± 0.13	0.00 ± 0.00	4.75 ± 0.62	-	-	4.48 ± 1.22
RD M1	1.88 ± 0.24	1.73 ± 0.28	1.53 ± 0.19	1.55 ± 0.26	2.55 ± 1.07	5.68 ± 0.91	10.12 ± 0.53	4.82 ± 0.28
RM M1	1.83 ± 0.36	1.85 ± 0.21	1.68 ± 0.40	1.63 ± 0.41	2.15 ± 0.38	6.00 ± 0.89	9.98 ± 0.61	5.15 ± 0.39
RD PM2	1.85 ± 0.37	1.80 ± 0.24	1.75 ± 0.44	1.45 ± 0.24	2.03 ± 0.33	5.78 ± 0.85	9.68 ± 0.55	5.02 ± 0.48
RM PM2	2.13 ± 0.34	1.70 ± 0.22	1.80 ± 0.32	1.68 ± 0.55	2.38 ± 0.94	5.60 ± 1.06	10.10 ± 0.66	5.20 ± 0.30
RD PM1	2.20 ± 0.29	1.73 ± 0.26	1.70 ± 0.41	1.38 ± 0.35	1.85 ± 0.31	5.90 ± 0.27	9.96 ± 0.50	4.83 ± 0.47
RM PM1	2.34 ± 0.45	1.78 ± 0.29	1.38 ± 0.13	1.05 ± 0.17	1.55 ± 0.47	6.06 ± 0.94	9.95 ± 0.69	4.25 ± 0.17
F ment	0.79 ± 0.25	0.80 ± 0.22	0.00 ± 0.00	3.80 ± 0.59	4.61 ± 0.74	4.70 ± 0.53	10.10 ± 0.26	4.63 ± 0.57

F mand: foramen mandibulare; RD M1: distal root of first molar; RM M1: mesial root of first molar; RD PM2: distal root of second premolar; RM PM2: mesial root of second premolar; RD PM1: distal root of first premolar; RM PM1: mesial root of first premolar; F ment: rostral foramen mentale; SD: standard deviation.

**Table 2:** Mandibular canal measurement values of male Van cats (*n* = 7).

Measurements	h	w	Fw	Flin	Bh	dep	Mh	Mw
References	Mean ± SD (mm)	Mean ± SD (mm)	Mean ± SD (mm)	Mean ± SD (mm)	Mean ± SD (mm)	Mean ± SD (mm)	Mean ± SD (mm)	Mean ± SD (mm)
Right Mandibular Canal								
F mand	2.43 ± 0.30	2.23 ± 0.14	2.25 ± 0.18	0.00 ± 0.00	5.18 ± 1.31	-	-	4.49 ± 1.72
RD M1	2.03 ± 0.54	1.88 ± 0.56	1.58 ± 0.32	1.58 ± 0.39	2.38 ± 0.57	5.98 ± 0.59	10.40 ± 0.72	5.06 ± 0.58
RM M1	1.85 ± 0.37	1.60 ± 0.08	1.85 ± 0.24	1.65 ± 0.26	2.35 ± 0.31	5.90 ± 0.32	10.10 ± 1.15	5.12 ± 0.36
RD PM2	1.80 ± 0.28	1.55 ± 0.17	1.93 ± 0.22	1.80 ± 0.51	2.53 ± 0.24	5.88 ± 0.81	10.23 ± 0.43	5.30 ± 0.51
RM PM2	1.68 ± 0.24	1.48 ± 0.17	1.90 ± 0.22	2.03 ± 0.83	2.53 ± 0.13	5.88 ± 0.61	10.12 ± 0.43	5.43 ± 0.38
RD PM1	1.98 ± 0.31	1.68 ± 0.29	1.48 ± 0.05	1.78 ± 0.67	2.38 ± 0.25	5.08 ± 0.74	9.45 ± 0.66	5.25 ± 0.99
RM PM1	2.00 ± 0.74	1.70 ± 0.27	1.32 ± 0.33	1.34 ± 0.69	2.15 ± 0.40	6.00 ± 0.46	10.16 ± 0.76	4.36 ± 0.64
F ment	0.79 ± 0.22	0.80 ± 0.25	0.00 ± 0.00	3.63 ± 0.78	4.73 ± 0.51	4.73 ± 0.90	10.25 ± 1.15	4.44 ± 0.59
Left Mandibular Canal								
F mand	2.50 ± 0.41	2.28 ± 0.17	2.25 ± 0.18	0.00 ± 0.00	5.20 ± 1.28	-	-	4.53 ± 1.76
RD M1	2.03 ± 0.54	1.80 ± 0.54	1.68 ± 0.26	1.33 ± 0.13	2.40 ± 0.54	5.93 ± 0.38	10.38 ± 0.50	4.83 ± 0.53
RM M1	1.83 ± 0.39	1.53 ± 0.15	1.85 ± 0.17	1.90 ± 0.42	2.40 ± 0.38	6.05 ± 0.61	9.84 ± 0.74	5.30 ± 0.27
RD PM2	1.93 ± 0.25	1.45 ± 0.13	1.88 ± 0.45	1.78 ± 0.64	2.60 ± 0.24	5.38 ± 0.99	9.93 ± 0.35	5.13 ± 0.46
RM PM2	1.80 ± 0.27	1.58 ± 0.24	1.95 ± 0.31	1.80 ± 0.37	2.63 ± 0.14	5.89 ± 0.57	10.33 ± 0.41	5.33 ± 0.33
RD PM1	2.08 ± 0.30	1.55 ± 0.34	1.63 ± 0.39	1.60 ± 0.81	2.58 ± 0.46	5.40 ± 0.89	10.07 ± 0.78	4.80 ± 0.92
RM PM1	1.98 ± 0.76	1.70 ± 0.27	1.30 ± 0.32	1.30 ± 0.62	2.38 ± 0.49	5.75 ± 0.50	10.12 ± 0.30	4.31 ± 0.50
F ment	0.79 ± 0.34	0.80 ± 0.32	0.00 ± 0.00	3.60 ± 0.74	4.73 ± 0.52	4.71 ± 0.87	10.23 ± 1.37	4.43 ± 0.66

F mand: foramen mandibulare; RD M1: distal root of first molar; RM M1: mesial root of first molar; RD PM2: distal root of second premolar; RM PM2: mesial root of second premolar; RD PM1: distal root of first premolar; RM PM1: mesial root of first premolar; F ment: rostral foramen mentale; SD: standard deviation.

**Table 3:** Statistical Evaluation (*p*-values) of Differences Between the Right and Left Sides in Van Cats.

	*p*-Values							
Measurements	h	w	Fw	Flin	Bh	dep	Mh	Mw
References	Female (*n* = 7)							
F mand	0.698	0.430	0.673	1.000	0.831	-	-	0.852
RD M1	0.691	0.494	0.192	0.704	0.901	0.787	0.825	0.113
RM M1	0.664	0.727	0.669	0.731	0.540	0.886	0.534	0.387
RD PM2	0.722	0.580	0.932	0.595	0.908	0.860	0.625	0.515
RM PM2	0.698	0.704	0.694	1.000	0.919	0.900	0.600	0.403
RD PM1	0.735	0.740	0.819	0.785	0.708	0.725	0.532	0.565
RM PM1	0.966	0.907	0.786	0.829	0.835	0.983	0.893	1.000
F ment	0.934	0.936	1.000	0.762	0.919	0.860	0.603	0.735
	Male (*n* = 7)							
F mand	0.722	0.559	1.000	1.000	0.977	-	-	0.966
RD M1	1.000	0.790	0.533	0.134	0.947	0.854	0.953	0.454
RM M1	0.923	0.297	0.998	0.205	0.792	0.575	0.624	0.311
RD PM2	0.378	0.240	0.796	0.950	0.595	0.321	0.178	0.525
RM PM2	0.397	0.386	0.734	0.516	0.191	0.975	0.368	0.609
RD PM1	0.551	0.456	0.333	0.659	0.332	0.479	0.134	0.396
RM PM1	0.961	1.000	0.910	0.911	0.355	0.349	0.899	0.873
F ment	1.000	1.000	1.000	0.942	1.000	0.967	0.977	0.977

F mand: foramen mandibulare; RD M1: distal root of first molar; RM M1: mesial root of first molar; RD PM2: distal root of second premolar; RM PM2: mesial root of second premolar; RD PM1: distal root of first premolar; RM PM1: mesial root of first premolar; F ment: rostral foramen mentale.

**Table 4:** Statistical comparison of mandibular canal measurements according to sex.

Measurement Areas/References	Female Mean ± SD (*n* = 7) (mm)	Male Mean ± SD (*n* = 7) (mm)	*p*
RD PM1—Bh (Right)	1.78 ± 0.37	2.38 ± 0.25	0.004 **
RD PM1—Bh (Left)	1.85 ± 0.31	2.58 ± 0.46	0.005 **
RD PM2—Bh (Right)	2.05 ± 0.31	2.53 ± 0.24	0.007 **
RD PM2—Bh (Left)	2.03 ± 0.33	2.60 ± 0.24	0.003 **
RM PM1—Bh (Right)	1.50 ± 0.41	2.15 ± 0.40	0.011 *
RM PM1—Bh (Left)	1.55 ± 0.47	2.38 ± 0.49	0.007 **
RD PM1—dep (Right)	5.85 ± 0.25	5.08 ± 074	0.033 *
RD PM1—dep (Left)	5.90 ± 0.27	5.14 ± 0.89	0.043 *
All Tooth Regions (h, w, Fw, Flin, Mh, Mw)	Similar Distribution	Similar Distribution	*p* > 0.05

RD PM1: distal root of first premolar; RD PM2: distal root of second premolar; RM PM1: mesial root of first premolar; SD: standard deviation. ** *p*< 0.01 statistical significance value, * *p*< 0.05 statistical significance value.

## Data Availability

The data sets generated for this study are available on request to the corresponding author.

## Abbreviations

The following abbreviations are used in this manuscript:

BW	Body Weight
CBCT	Cone-Beam Computed Tomography
DICOM	Digital Imaging and Communications in Medicine
FOV	Field of View
i.m.	Intramuscular
MC	Mandibular Canal
MPR	Multiplanar Reconstruction
SPSS	Statistical Package for the Social Sciences
